# Non-referral of potential organ donors in South Africa: insights, challenges and ethical dilemmas

**DOI:** 10.11604/pamj.2018.29.223.14756

**Published:** 2018-04-24

**Authors:** Poobalan Naidoo, Harriet Rosanne Etheredge, Virendra Rambiritch, Akira Singh, Scott Mahoney, Vanesha Naidu

**Affiliations:** 1 King Edward VIII Tertiary Hospital, Department of Internal Medicine, South Africa; 2 Wits Donald Gordon Medical Centre, Johannesburg and Department of Internal Medicine, Faculty of Health Sciences, University of the Witwatersrand, Johannesburg, South Africa; 3 University of KwaZulu-Natal, Biomedical Research Ethics Committee, KwaZulu-Natal, South Africa; 4 King Edward VIII Tertiary Hospital, Department of Radiology, South Africa

**Keywords:** Organ donation, organ transplantation, ethics

## Abstract

Traditionally, minimal potential organ donor referrals emanate from general medicine departments. We use a clinical vignette to draw attention to challenges related to referral of potential organ donors from general internal medicine departments. In addition, we provide potential solutions to overcome challenges and reflect on the ethical issues of non-referral of potential organ donors. It is hoped that this paper will increase the awareness of organ donation in the medical fraternity in Africa and thus mitigate critical shortages of organs for transplantation.

## Perspectives

**Case vignette**: A 15 year old Caucasian male, with a complaint of headaches in the preceding two weeks, suddenly collapsed with a loss of consciousness while exercising at gym. The nature of the exercise that he was engaged in when he collapsed is unknown. The aforementioned headaches had been reported to a general practitioner. As it was not associated with any neurological symptoms, it was managed as a simple headache with analgesia (unknown name) and not investigated further. He received immediate cardiopulmonary resuscitation (CPR) by a trained provider and had return of spontaneous circulation (ROSC) after an unknown period of time. He was intubated and ventilated at the scene and transferred by an advanced life support team to King Edward Hospital VIII Tertiary Hospital. On arrival to the Acute Medical Unit he was hypotensive with a blood pressure of 91/31 mmHg on inotropic support. He had a sinus tachycardia of 130 beats/minute and was saturating at 100 percent on 100 percent oxygen with an arterial partial pressure of oxygen of 29.6 kPa. An arterial blood gas analysis showed a metabolic acidosis (pH 7.19, lactate 4.7 mmol/l and a standard bicarbonate of 20.2 mmol/l). He was normoglycaemic (glucose 10.8 mmol/l) with a Glasgow coma scale (GCS) of E1M1 tubed (1 for motor score, 1 for eye opening and the verbal score could not be determined because the patient was intubated). Both pupils were fixed, dilated and non-responsive to light and the corneal reflex was absent. Fundoscopy revealed bilateral retinal haemorrhages. Given the presentation of severe preceding headaches followed by a sudden collapse while exercising and ensuing non-responsive coma in a previously healthy man the primary consideration was that of a catastrophic intracranial haemorrhage.

**Figure 1:**
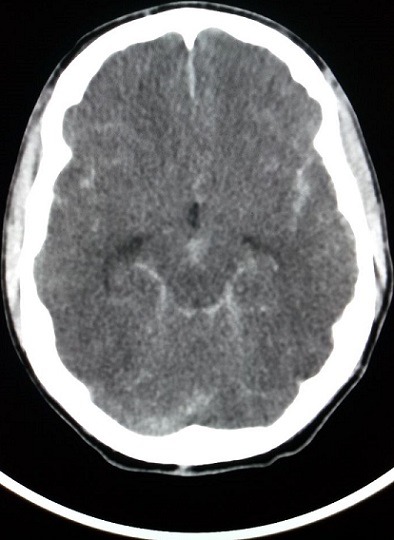
Unenhanced CT axial image demonstrates extensive subarachnoid haemorrhage with blood filling the central basal cisterns, bilateral Sylvian fissures and the anterior inter-hemispheric fissure with associated early hydrocephalus

**Figure 2:**
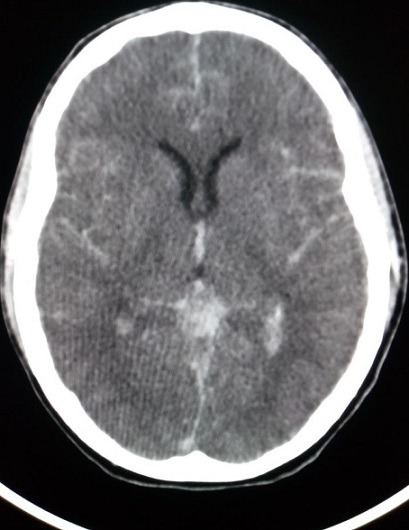
Unenhanced CT axial image shows subarachnoid blood with intra-ventricular extension and peripheral hemorrhage outlining the cerebral sulci

This was confirmed on non-contrast computerized tomography (CT) scan, which demonstrated diffuse acute subarachnoid haemorrhage with blood present in the basal cisterns, ventricular system and peripheral extra-axial spaces (Figure 1, Figure 2). Although CT angiography was not performed or a sentinel bleed identified, the most likely aetiology was that of subarachnoid haemorrhage secondary to an underlying cerebral vascular abnormality, likely a berry aneurysm. A neurosurgery consult found the patient unsuitable for neurosurgical intervention because the bleed was too extensive to be amenable to drainage and brainstem reflexes were absent. The patient subsequently went into asystole and regained spontaneous circulation after 5 minutes of CPR. The family was counselled on the likely diagnosis of subarachnoid haemorrhage and poor prognosis. The family immediately requested for him to be considered for organ donation and the transplant coordinator was informed. It took approximately six hours for the patient to be transferred to an intensive care unit in a hospital with the necessary infrastructure and resources, where a transplant team might successfully harvest his organs. During the 6 hour period, the patient required close monitoring, increasing inotropic support and fluid therapy to maintain adequate perfusion and oxygenation. The sudden and unexpected demise of an otherwise healthy young student, received extensive press coverage. An off shoot of this tragic event was public awareness which promoted the merits of organ donation and thereby potentially increasing the donor pool. This case was presented at an internal medicine grand round, further serving to disseminate knowledge to clinicians who have the potential to refer patients to organ transplant coordinators.

South Africa's first solid organ transplant was a kidney transplant in 1966 which was performed by Prof Thomas Starzl, a US-based transplant surgeon, and Prof Bert Myburgh, a South African. This was followed a year later with Professor Christiaan Barnard and his team at Groote Schuur Hospital, who performed the first human heart transplant [1]. More recently a team from the University of Stellenbosch performed the world's first successful penis transplant [2]. In addition to the aforementioned pioneering transplants, Muller et al [3], have contributed significantly to the scientific literature around outcomes of HIV positive patients receiving kidneys from HIV positive donors. The Wits Donald Gordon Medical Centre runs the first living donor liver programme in South Africa. Despite the proud transplant history of South Africa, the transplant program for solid organs in the state sector in KwaZulu-Natal (province of index case), is limited to renal transplant surgery. On average there are only 8 transplants per year despite the 436 patients on the waiting list, with numbers increasing by 30% each year (data on file). Additionally, very few donors are deceased donors. This is in contrast to almost all other centres with successful transplant programmes. Furthermore, in KwaZulu-Natal transplants after circulatory/cardiac death and HIV positive to HIV positive renal transplants is not performed, despite the pioneering work of Cape Town colleagues. South Africa has not been excluded from controversies around organ transplantation. The “kidney gate” saga led to the arrest of South African doctors for allegedly transplanting kidneys involving Israeli and Brazilian citizens [4]. The charges against the doctors were eventually withdrawn and the hospital group involved was fined [5]. Currently, in South Africa, the major transplant units are based in Cape Town and Johannesburg. In 2015, 369 solid organ transplants were carried out in South Africa, with the majority (251) being kidney transplants [6].

**Organ shortage**: Despite the urgent and increasing need for life saving organ transplants, the number of organ donors remains low. The majority of the organs are from living donors who have an emotional or genetic link to the recipient. Traditionally organ donors have been patients fulfilling neurological criteria used to declare “brain death” [7]. Given the shortage of organs there is now a shift and a greater use internationally of organs from individuals after cardiac death ie, death declared on the basis of cardiopulmonary criteria. In Southern Africa and particularly in South Africa the devastating HIV epidemic has reduced the potential organ donor pool. To a limited extent, as seen with the kidney transplantation between HIV positive patients, there has been some movement to address this challenge. However, both the number and frequency of these transplants remain relatively small. Furthermore, the growing shortage of organ donors has resulted in an increasing effort to use less optimal organs i.e, marginal donors/expanded criteria donors with the selection criteria for solid organ donors becoming less restrictive [8, 9]. South Africa lags behind the international community in using more organ donors after cardiac death. This is unfortunate as it is a potential source of increased referrals of organ donors to organ transplant coordinators. Internal medicine departments are more likely to be involved with patients with cardiac death and this may serve as a rich source of referrals.

**Declining number of referrals**: Although the demand for organs has increased, the number of referrals of potential organ donors has been static. A small unpublished study at Groote Schuur Hospital [8] showed that a significant number of potential organ donors were not referred to organ transplant coordinators. Given that this study was done at a relatively well staffed and resourced quaternary hospital, the situation in other hospitals may be even worse because of a major limitation in human and financial resources. There is thus a high probability that more potential organ donors can be referred to organ transplant coordinators if medical practitioners and nurses are sensitized to the organ transplantation process. While King Edward VIII is a tertiary hospital with large surgical, medical and intensive care departments, the referral rate is very low-only 3 patients were referred from King Edward VIII Hospital Trauma Unit since 2006 and no referrals from Medicine/intensive care unit. The reasons for this low referral rate is speculative and may include lack of awareness or lack of willingness to refer.

**Challenges to referring potential organ donors from internal medicine departments**: It appears then there are two major challenges that limit referral from internal medicine departments to organ transplant coordinators i.e, limited education and awareness together with the lack of a co-ordinated response team with the infrastructure to support this process.

**Education and Awareness**: Sobnach et al [10] evaluated the attitudes and beliefs of medical students with respect to organ donation, procurement and transplantation at the University of Cape Town. Of the 346 students surveyed, only 8% were registered organ donors. Alarmingly only 18% knew where to find information for potential donors and recipients. This study was published approximately 5 years ago and the current perceptions of medical students is unknown. Furthermore, the study only evaluated students' perceptions at a single South African University and thus may not be applicable to the entire country. We, the authors of this paper, have direct and indirect exposure to medical curricula, medical students and medical interns from various medical schools in South Africa, and are of the opinion that the Sobnach et al [10] study is in line with what is found at other Universities, and the results of the studies from other Universities may fair less well, given that the University of Cape Town is a leading University in South Africa and Africa. There have been medical curriculum changes to inform students of organ donation. However, curriculum inclusion of organ donation is not the norm and there remain settings were no formal introduction to organ donation is done, which limits the referral of patients to organ donor coordinators. It is recommended that there should be national standardisation of the exposure to organ donation, procurement and transplantation with integration of interactive teaching sessions with transplant surgeons, transplant coordinators and other relevant stakeholders. Medical interns are seldom exposed to organ donation and transplantation during training. It is less probable that doctors will refer patients to organ transplant coordinators if they are unaware that such a system exists. Perhaps internal medicine consultants, who have been exposed to organ transplantation, should educate junior medical registrars, medical officers and interns on the importance of identifying potential candidates for organ transplantation and highlight the process to ensure adequate referral.

**Co-ordinated response team**: Calls in medicine are busy with many acutely ill patients and numerous consults for inpatients in obstetric and gynaecological, orthopaedic and surgical wards. Managing a potential organ donor before transfer to an intensive care unit is time consuming and draws attention from the management of other sick patients. Furthermore, it is emotionally and psychologically challenging to manage the families of these patients, which further increases the demands on an already stretched team. A solution may be that if the on call team is severely strained during a busy call, the medical consultant should lead the process and physically be at the hospital to ensure adequate care of the patient before transfer. Other resource limitations included limited number of organ donor co-ordinators and availability of skilled personnel.

**Ethical considerations**: Organ donation and transplant in SA is imbued with ethical issues. These are shaped by the context in which health care is provided and then borne out in the clinical system where transplant referrals and the process of consenting to organ donation take place [11]. In terms of context, there appears to be a distrust of transplant amongst the SA population. To some extent this could be traced back to colonial medical systems [12] but there is little doubt the context has also been influenced by events like the “kidney gate case” and the manner in which it was handled in the media [11]. Context is also shaped, to a large extent, by personal beliefs, religious and socio-cultural practices, all of which have a bearing on the clinical situation in which consent for organ donation might be sought. At a hospital level, for instance in the vignette described at the beginning of this paper, ethical issues manifest themselves in the conversations that health professionals have with donor families. These too are shaped by context and experiences. For instance, health professionals who have a negative perception of organ donation may feel unwilling to consider referring a brain stem death (BSD) patient (or a patient nearing BSD) [13]. Similarly, many health professionals feel uncomfortable discussing end-of-life issues and organ donation with families, and thus they avoid these conversations. This has implications for the exercise of autonomy, where families of BSD patients should be given all options available to them, and make an informed decision on this basis [13]. Whether or not clinicians have an ethical duty to refer patients is unclear, and in South Africa the situation is trickier because there is no nationally endorsed, robust referral policy. The lack of guidance can result in health professionals feeling insecure about their roles in facilitating organ donation, and they may also feel unsupported should they choose to refer. Certainly, it has been argued that a mandatory referral process is ethical and should be carefully legislated [13], and this would likely lead to an increase in donor numbers-an outcome in the best interests of recipients and also, in some cases, a means for donor families to gain closure [14, 15]. However, it is vital to ensure that any measures which aim to increase the number of organ donors through by shoring-up autonomy and beneficence are also framed within justice principles [16]. This would involve a commitment to expand transplant resources, increase the number of transplant centres (especially in the state sector), facilitate better access for state patients and increase the scope of transplant programmes available to state patients. Justice principles have a basis in equality, stating that people must be treated equally. Any transplant intervention which creates an increase in donor numbers, but does not ensure corresponding equitable accessed across the population, may not be just in SA.

## Conclusion

The non-referral of potential organ donors has societal and ethical ramifications. General internal medicine teams can increase the number of referrals to organ transplant coordinators. Consultant physicians have the knowledge and expertise to lead the referral of potential organ donors to transplant coordinators.

## Competing interests

The authors declare no competing interest.

## References

[1] Barnard CN (1967). The operation: a human cardiac transplant: an interim report of a successful operation performed at Groote Schuur Hospital Cape Town. SAMJ.

[2] Merwe AV, Zarrabi A, Zuhlke A, Barsdorf N, Moosa R (2017). Lessons learned from the world's first successful penis allotransplantation. J Mater Sci Mater Med.

[3] Muller E, Barday Z, Mendelson M, Kahn D (2015). HIV-positive-to-HIV-positive kidney transplantation-results at 3 to 5 years. N Eng J Med.

[4] Sidley P (2005). South African doctors arrested in kidney sale scandal. BMJ: British Medical Journal.

[5] Allain J (2011). Trafficking of persons for the removal of organs and the admission of guilt of a South African hospital. Med Law Rev.

[6] Organ donation foundation

[7] Steinbrook R (2007). Organ donation after cardiac death. N Eng J Med.

[8] Muller E (2013). Organ donation and transplantation in South Africa-an update. Continuing Medical Education.

[9] Tullius SG, Volk HD, Neuhaus P (2001). Transplantation of organs from marginal donors. Transplantation.

[10] Sobnach S, Borkum M, Millar AJ, Hoffman R, Muller E, McCurdie F, Kahn D (2012). Attitudes and beliefs of South African medical students toward organ transplantation. Clin Transplant.

[11] Etheredge HR (2015). "Hey sister! Where's my kidney?"- exploring ethics and communication in organ transplantation in Gauteng Province, South Africa.

[12] Vaughan M (1991). Curing Their Ills: Colonial Power and African Illness.

[13] Etheredge H, Penn C, Watermeyer J (2017). "Opt-in or Opt-out to Increase Organ Donation in South Africa: appraising Proposed Strategies Using an Empirical Ethics Analysis.". Dev World Bioeth.

[14] Chandler JA, Connors M, Holland G, Shemie SD (2017). "Effective" Requesting: a Scoping Review of the Literature on Asking Families to Consent to Organ and Tissue Donation. Transplantation.

[15] Merchant SJ, Yoshida EM, Lee TK, Richardson P, Karlsbjerg KM, Cheung E (2008). Exploring the psychological effects of deceased organ donation on the families of the organ donors. Clin Transplant.

[16] Beauchamp TL, James FC (2001). Principles of Biomedical Ethics: Oxford, Oxford University Press.

